# Protective Effects of Isolated Curcumin From *Curcuma longa* on Key Enzymes Involved in the Insulin Signaling Pathway and Digestive and Metabolic Enzymes Associated With Obesity, Type 2 Diabetes, and Hypertension

**DOI:** 10.1155/jdr/8050374

**Published:** 2025-05-08

**Authors:** Munirah S. O. Alhar, Walaa I. El-Sofany, Aljazi Abdullah AlRashidi, Khaled Hamden

**Affiliations:** ^1^Department of Chemistry, College of Science, University of Ha'il, Ha'il 81451, Saudi Arabia; ^2^Medical and Diagnostic Research Center, University of Ha'il, Ha'il 55473, Saudi Arabia; ^3^Biolival Laboratory, Higher Institute of Biotechnology of Monastir, Monastir University, Monastir, Tunisia; ^4^Higher School of Health Sciences and Technology of Sfax, Sfax University, Sfax, Tunisia

**Keywords:** curcumin, diabetes, insulin signalization pathway, key enzyme, obesity

## Abstract

This study explores the potential of curcumin (CUR), extracted from *Curcuma longa*, in combating obesity and Type 2 diabetes. Obesity and Type 2 diabetes were induced in rats through a high-fat and high-fructose diet (HFFD), and CUR, after purification and characterization by Fourier transform infrared spectroscopy (FTIR) and ultraviolet (UV) spectroscopy, was administered for 3 months via gastric gavage. The results show that CUR supplementation activates the insulin signaling pathway in a dose-dependent manner, leading to improved insulin sensitivity. Specifically, administering CUR at a daily dose of 100 mg/kg significantly reduces the activities of protein tyrosine phosphatase (PTP1B) and dipeptidyl peptidase-4 (DPP-4) by 43% and 45%, respectively, in obese and Type 2 diabetic rats compared to untreated obese rats. Furthermore, CUR effectively inhibits lipase and *α*-amylase activities at both the serum and intestinal levels. In obese rats, CUR administration reduces glycogen phosphorylase (GP) activity by 35% and enhances glycogen synthase (GS) activity by 78%, leading to a substantial increase in hepatic glycogen content. Additionally, CUR also led to a 21% reduction in food intake and a 12% decrease in water consumption. These changes contributed to significant reductions in the blood sugar and glycosylated hemoglobin (HbA1c) levels, with decreases of 59% and 53%, respectively. Additionally, administering CUR at a dose of 100 mg/kg body weight reduced thiobarbituric acid reactive substances (TBARSs), hydrogen peroxide (H_2_O_2_), and total oxidant status (TOS) in obese and diabetic rats, with reductions of 49%, 59%, and 58%, respectively. Furthermore, CUR demonstrates a strong regulatory effect on the levels of low-density lipoprotein cholesterol (LDL-C), high-density lipoprotein cholesterol (HDL-C), and total cholesterol (TC). Overall, these results underscore the CUR potential for treating and preventing diabetes and obesity.

## 1. Introduction

The International Diabetes Federation projected that 10.5% of the global adult population, equivalent to 537 million people aged 20–79, would have diabetes [1]. It is projected that by 2030, the number of people with diabetes mellitus (DM) will reach 643 million, and by 2045, it will increase to 783 million. This represents a 46% rise in the prevalence of DM, despite a projected 20% increase in the world's population. Type 2 diabetes mellitus (T2DM) is among the most serious chronic health conditions. Recently, the prevalence of diabetes has surged by 69% in developing countries, while in developed nations, it is expected to rise by 20% between 2010 and 2030 [2]. Furthermore, the cost of diabetes care is projected to reach approximately US$ 1.7 trillion between 2011 and 2030. This financial burden is exacerbated by the expectation that the majority of new cases will arise in low- and middle-income countries [3, 4]. The Arab Gulf countries are among the regions with the highest prevalence of diabetes, with rates ranging from 8% to 22%, according to region-specific data from the International Diabetes Federation. In Saudi Arabia, the significant rise in diabetes cases has been attributed to rapid epidemiological transitions, urbanization, unhealthy diets, and decreased physical activity in recent decades [5]. Individuals with diabetes are at increased risk of severe complications, including hyperlipidemia, hypertension, and liver and kidney toxicity [6–8].

A therapeutic approach to mitigating Type 2 diabetes and obesity involves reducing glucose and lipid absorption by inhibiting carbohydrate- and lipid-hydrolyzing enzymes, such as *α*-amylase and lipase, in the digestive system [9]. Acarbose, an inhibitor of intestinal *α*-glucosidases and pancreatic *α*-amylase, slows carbohydrate digestion and limits postprandial hyperglycemia without causing hypoglycemia. By delaying the conversion of starches and disaccharides into glucose, it enhances insulin sensitivity, reduces glycemic spikes, and lowers the risk of metabolic complications, including postprandial triglyceride accumulation. Furthermore, by limiting carbohydrate absorption, acarbose may promote slight weight loss in some patients. However, its use can be associated with digestive side effects such as bloating and flatulence, due to the fermentation of unabsorbed carbohydrates in the intestine.

Furthermore, the activation of insulin signaling by inhibiting dipeptidyl peptidase-4 (DPP-4) and tyrosine phosphatase 1B (PTP1B) plays a crucial role in improving glucose metabolism. DPP-4 is an enzyme that degrades incretin hormones [10, 11], such as glucagon-like peptide-1 (GLP-1), which stimulate insulin release after meals. By inhibiting DPP-4, active GLP-1 levels increase, which promotes insulin secretion from the pancreas and reduces glucagon secretion, thereby lowering blood glucose levels, especially after meals [12–15]. However, synthetic inhibitors of these enzymes, while effective, lead to various side effects. GLP-1 agonists, such as tirzepatide, and SGLT2 inhibitors, as empagliflozin, cause side effects, including nausea, vomiting, abdominal pain, and an increased risk of pancreatitis for GLP-1 agonists [16]. SGLT2 inhibitors are associated with an increased risk of urinary infections, dehydration, hypotension, and diabetic ketoacidosis [17]. In light of these negative effects of synthetic compounds, the search for natural substances with inhibitory effects on digestive enzymes, as well as inducing insulin signaling through the inhibition of DPP-4 and PTP1B, appears promising for improving diabetes without side effects. Additionally, these natural substances could offer various positive effects, such as antioxidant and anti-inflammatory activities, contributing to a safer and more comprehensive management of diabetes [7].

Consequently, recent research has focused on identifying effective natural inhibitors of *α*-amylase and lipase. There is a pressing need for alternative remedies, as evidenced by previous studies indicating the efficacy of various plants in diabetes treatment with minimal side effects [18, 19]. Medicinal plants offer promising, safe alternatives for treating inflammation, hyperglycemia, obesity, and hypertension [20–23], attracting attention due to their efficacy, minimal side effects, and relatively low cost.

Curcumin (CUR), a polyphenol extracted from the *Curcuma longa* plant, has gained recognition as a promising remedy for a range of ailments, including cancer, infections, and dementia [24–26]. CUR, derived from *Curcuma longa* (commonly known as turmeric), is used as a spice, natural food coloring, and dietary supplement. It exhibits a range of preventive and therapeutic effects against various disorders and diseases, including inflammatory, metabolic, neurological, and carcinogenic conditions [27–32].

This study aims to isolate and refine CUR from *Curcuma longa*; examine the stability of this compound concerning pH, temperature, and storage conditions; and analyze its effects on digestive and metabolic enzymes in obese rats. This investigation represents a novel exploration as such assessments have not been conducted previously.

## 2. Materials and Methods

### 2.1. CUR Isolation

Rhizome samples of *Curcuma longa* were purchased from a local supermarket, subsequently identified by Dr. Fatma Guesmi, a botanist and Assistant Professor at the Faculty of Sciences of Gafsa, and then ground using a mixer. The dried powdered rhizome sample of *Curcuma longa* (100 g) was extracted with petroleum ether (250 mL) for 3 days at room temperature (three times). The mixture was then filtered using filter paper. The remaining residue was extracted with ethanol (200 mL) for 3 days at room temperature (three times). This mixture was also filtered with filter paper, and then, the resulting filtrate was evaporated using a rotary evaporator to reduce volume and then achieve complete dryness. The resulting crude extract was weighed. The crude extract (0.21 g) was then subjected to column chromatography. The entire extract was dissolved in chloroform and thoroughly adsorbed onto silica gel. The adsorbed material, after drying, was transferred to a column packed with 20 g of gel in chloroform. The column was then eluted consecutively with chloroform: methanol (99:1, 600 mL). Each fraction collected was monitored by thin-layer chromatography (TLC) using chloroform: methanol (99:1) as the solvent system. The spots on the TLC were visualized by spraying with a 5% ferric chloride solution. The isolated compound was identified using FTIR and UV-visible spectroscopy. The infrared spectrum of the isolated compound was recorded and examined to determine whether the respective functional groups were present, using an FTIR spectrophotometer. Additionally, for the identification of the isolated compound, the ultraviolet absorption spectrum was recorded on a Shimadzu UV-240 UV-visible spectrophotometer.

### 2.2. Animals

Male adult Wistar rats, 8 weeks old and 163 g ± 9 in weight, were obtained from the Animal Experimental Laboratory. The laboratory was maintained at a temperature of 23 ± 2°C and approximately 55% humidity. Prior to the trials, the rats were allowed to acclimate to their new environment for 7 days, with access to a normal diet and unlimited water. They had full access to water for 24 h before the trials began and were then deprived of water for 1 hour. The rats were considered obese if their body weight increased by more than 20% and there was a significant elevation in blood glucose levels [33, 34].

### 2.3. Experimental Study

In this study, a total of 35 male were used. The rat experiments were conducted in compliance with the National Institutes of Health's “Guide for the Care and Use of Laboratory Animals” (NIH Publication No. 85-23, revised 1996) and the recommendations of the Animal Care and Use Commission (approval numbers: CEEA-ENMV 28/04 and CER-SVS 0038/20222027-0219). After a week of acclimatization on a standard diet comprising corn, soy, and vitamins provided by the Company of Animal Nutrition [6], obesity was induced using a hypercaloric high-fat and high-fructose diet (HFFD), which contained 0.1% cholic acid, 12.5% sheep fat, 12.5% fructose, and 75% conventional diet [6]. The groups were organized as follows: Group 1 consisted of normal control rats fed a standard diet, referred to as (Con). Group 2 included rats that received the HFFD to induce obesity associated with Type 2 diabetes, referred to as (O). Groups 3, 4, and 5 included obese rats treated with CUR at doses of 50, 100, and 150 mg/kg body weight daily for 3 months via gastric gavage and referred to as O + CUR_50_, O + CUR_100_, and O + CUR_150_, respectively. Additionally, Groups 6 and 7 consisted of obese rats with Type 2 diabetes, which were treated, respectively, with a combination of CUR100 and glucor named (O-CUR_100_-Gluc) or with glucor (O-Gluc) alone at a dose of 10 mg/kg [35] body weight via gastric gavage. At the end of the experiment, the rats were decapitated, and trunk blood was collected. The serum was prepared by centrifugation (1500 × *g*, 15 min at 4°C). The pancreas was excised, and all samples were stored at −80°C for further analysis.

### 2.4. Analytical Methods

The small intestine was excised from each rat, rinsed with 0.9% NaCl, and homogenized. After centrifugation at 5000 × *g* for 15 min, the supernatant was frozen for enzymatic assays. Pancreas lipase activity was measured using BIOLABO kits (France, ref. 99223) by assessing fatty acid release from 1,2-diglycerides. *α*-Amylase activity was evaluated using the CNPG3 substrate (France, ref. 99881). The maltase and sucrase activities in the rats in the small intestine were determined by incubating the pancreatic extract from each rat with their respective substrates, maltose and sucrose, at 37°C. After incubation, the addition of DNS (dinitrosalicylic acid) resulted in the formation of a red–orange color, with the maximum absorbance measured at a wavelength of 540 nm [36, 37]. Blood samples were centrifuged at 3000 × *g* for 15 min. Glucose levels were determined using the glucose oxidase method (Kit Biomaghreb, ref. 26019), and LDH activity was measured by the conversion of pyruvate and NADH into lactate and NAD+ (Kit Biolabo, ref K4011). Serum levels of total HbA and HbA1c were assessed with commercial kits (Kit BIOLABO, France, ref. K2010 and K3502200) [38]. Total cholesterol (TC), low-density lipoprotein cholesterol (LDL-C), and high-density lipoprotein cholesterol (HDL-C) were measured with Biomaghreb kits (Refs. 20111, 20113, and 201132). PTP1B activity was assessed using Sigma's P6244-50UG and p-nitrophenyl phosphate (p-NPP), with an absorbance measured at 405 nm [39]. DPP-4 activity and inhibition by CUR were determined by monitoring 4-nitroaniline release and measuring absorbance at 405 nm. The hydrogen peroxide (H_2_O_2_) level was assessed by oxidizing phenol red with horseradish peroxidase and measuring absorbance at 610 nm [40]. Serum total oxidant status (TOS) was measured using the RelAssay Diagnostics Kit (Ref. RL0024). A single gram of the intestinal mucosa or liver was homogenized in Tris-HCl buffer at a low temperature, followed by centrifugation at 3000 × *g* for 15 min. The supernatant was collected and stored at −80°C for further analysis. Protein levels were determined by spectrophotometry, analyzing the blue–violet complex formed when peptide bonds reacted with Cu^2+^ in an alkaline solution (Kit Biomaghreb, La Goulette, Tunisia, ref. 27016). The activities of hepatic glucose metabolism enzymes, including fructose-1,6-bisphosphatase (FBP), pyruvate kinase (PK), glucose-6-phosphatase (G6P), and hexokinase (HK), were measured using established techniques [31–34, 41]. The assays described by Cornblath et al. [42] and Leloir et al. [43] were used to measure liver glycogen content and the activity of hepatic glycogen synthase (GS) and glycogen phosphorylase (GP). Blood pressure and heart rate were measured using a pressure meter (Niprem 645, Cibertec, Madrid, Spain) and the pneumatic “tail-cuff” method. Thiobarbituric acid reactive substances (TBARSs) in the liver were assessed using the method outlined by Buege and Aust [44].

### 2.5. Statistical Analysis

StatView 5.0 was used for statistical analysis. Results are presented as means ± standard error of the mean (SEM). Group differences were assessed using the Fisher test, with significance set at *p* < 0.05.

## 3. Results

### 3.1. Identification of the Compound Isolated From the Crude Extract

From the silica gel column chromatographic separation of the crude (ethanol) extract of the rhizome of *Curcuma longa*, a compound was obtained as orange needles with a yield of 0.6248% based on the raw material. The retention factor values of the compound were determined to be 0.44 and 0.34 using chloroform:methanol (99:1 volume/volume) and petroleum ether:ethyl acetate (1:1 volume/volume) as solvent systems, respectively, and were consistent with those of authentic CUR. Additionally, the melting point of the isolated compound was recorded as 175°C, closely aligning with the literature value for CUR, which is 177°C. An iodine vapor test and a 5% ferric chloride test revealed a brown color, indicating the presence of phenolic OH groups characteristic of CUR. Based on these physicochemical properties, the compound was identified as CUR. Subsequently, the isolated compound was further characterized and identified using FTIR and UV-visible spectroscopy.

### 3.2. CUR Toxicity

In this study, rats administered with a dosage of 2 g/kg of CUR exhibited no indications of physiological toxicity or mortality.

### 3.3. CUR Identification of the Isolated Compounds by FTIR and UV-Visible Spectroscopy

**Figure 1:**
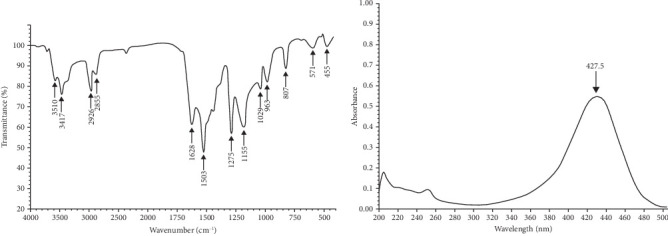
Fourier transform infrared (FTIR) and absorbance spectra of purified curcumin. Our results show a similarity with the literature, confirming that our compound is pure.

The FTIR spectrum revealed several key features: Broad bands between 3600 and 3317 cm^−1^ were attributed to the -OH stretching vibrations of phenolic hydroxyl groups. Aliphatic C–H stretching vibrations from CH₃ and CH₂ groups were observed at 2924 and 2854 cm^−1^, respectively. The carbonyl stretching vibration of the keto–enol system was detected at 1620 cm^−1^, while the C=C stretching vibrations in the aromatic ring system appeared at 1597, 1504, and 1427 cm^−1^. A strong and sharp signal around 1273 cm^−1^ indicated = CH in-plane deformation, with two additional strong signals near 1195 cm^−1^ corresponding to C=O bending. The symmetric and asymmetric stretching vibrations of the C–O–C group were observed at 1026 cm^−1^. Bending vibrations of trans olefinic C–H bonds were noted at 964 cm^−1^, and those of aromatic C–H bonds appeared at 864 cm^−1^ and 810 cm^−1^. The UV spectrum of CUR in ethanol showed a maximum absorption wavelength at 427.86 nm, indicating the presence of conjugated double bonds, which is in close agreement with the literature value of 422 nm (Figure 1).

### 3.4. CUR Ingestion, Lipase Activity, Body Weight, and Serum Lipid Levels in Obese Rats

**Figure 2:**
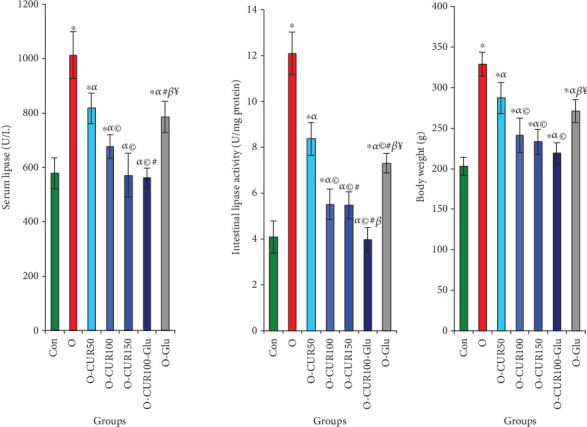
Evaluation of CUR ingestion on lipase activity in the small intestine and serum and body weight of obese rats. The results of this study demonstrate that the ingestion of CUR significantly inhibited the activities of all three enzymes, suggesting its potential role in regulating lipid digestion and absorption. The values are presented as mean ± SD for each group of five animals. Statistical significance is indicated as follows: ⁣^∗^*p* < 0.05 when comparing to controls; ^*α*^*p* < 0.05 when comparing to obese rats; ^©^*p* < 0.05 when comparing to obese rats treated with CUR_50_; ^#^*p* < 0.05 when comparing to obese rats treated with CUR_100_; ^*β*^*p* < 0.05 when comparing to obese rats treated with the combination CUR_150_; and ^¥^*p* < 0.05 when comparing to obese rats treated with the combination CUR_100_ and Glu.

Compared to normal rats, the intestinal and serum lipase activities in obese rats increased significantly by 202% and 75%, respectively. This increase in lipase activity was associated with a 62% rise in body weight. Elevated lipase activity also led to enhanced lipid digestion, which resulted in a 30% increase in blood TC and an 83% increase in LDL-C, while HDL-C decreased by 16%. In contrast, CUR treatment reduced lipase activity in obese rats in a dose-dependent manner. Specifically, administration of CUR at a dose of 100 mg/kg significantly decreased intestinal and serum lipase activities by 59% and 40%, respectively, leading to a 20.6% reduction in body weight. Furthermore, CUR treatment resulted in a 20% reduction in TC, a 39% reduction in LDL-C, and a 38% increase in HDL-C in the serum of obese rats (Figure 2, Table 1).

### 3.5. Effects of CUR Supplementation on Maltase, Sucrase, and *α*-Amylase Activities and Glycemic Control in Obese Rats

**Figure 3:**
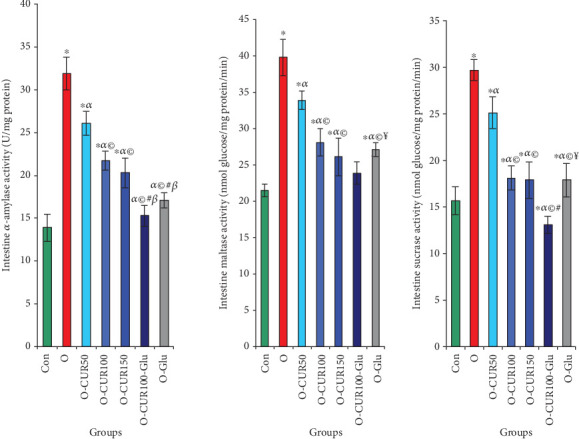
Effect of CUR ingestion on intestinal maltase, sucrase, and *α*-amylase activities of obese rats is depicted. Curcumin strongly inhibits the activity of polysaccharide hydrolysis, resulting in an antihyperglycemic effect. Statistical analyses are provided in Figure 2.

In obese rats, the intestinal activities of maltase, sucrase, and *α*-amylase increased significantly by 85%, 89%, and 129%, respectively, compared to normal rats. This rise in enzyme activity led to increases in blood glucose and HbA1c levels by 206% and 124%, respectively. Supplementation with CUR in obese rats inhibited the activities of these digestive enzymes in a dose-dependent manner. Specifically, administering CUR at 100 mg/kg reduced the maltase, sucrase, and *α*-amylase activities by 30%, 39%, and 32%, respectively. Consequently, blood glucose and HbA1c levels decreased by 42% and 43%, respectively, highlighting CUR significant antidiabetic effects (Figure 3).

### 3.6. Effects of CUR on Blood DPP-4 and PTP1B Activities in the Serum of Obese Rats

**Figure 4:**
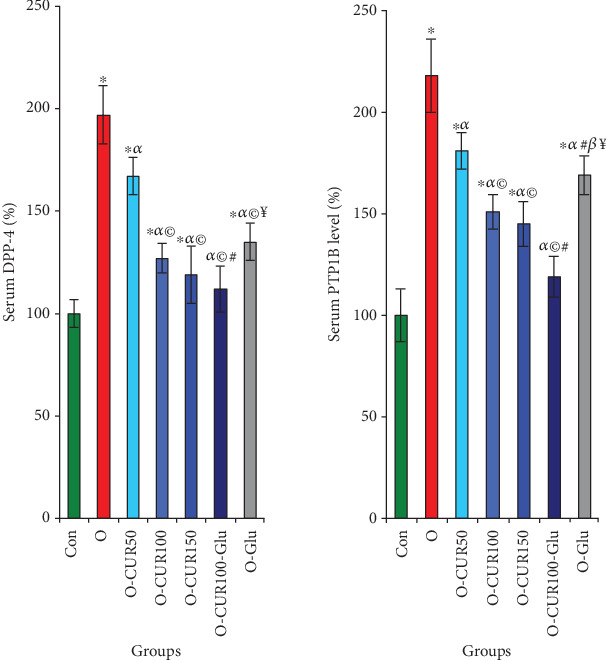
Effect of curcumin on key insulin signaling enzymes in rats with Type 2 diabetes, such as DPP-4 and PTP1B, was investigated. Our results demonstrate that their administration potentially inhibits the activity of DPP-4 and PTP1B, consequently affecting insulin signaling. Statistical analyses are provided in Figure 2.

The results of this study indicate that Type 2 diabetes or insulin resistance associated with obesity is linked to altered insulin signaling pathways, as evidenced by a significant increase in the activities of DPP-4 and PTP1B in the serum by 97% and 118%, respectively, compared to normal rats. Conversely, CUR supplementation potentially stimulated the insulin signaling pathway by suppressing the activities of these two enzymes by 30% and 35%, respectively, when administered at a dose of 100 mg/kg body weight for 90 days (Figure 4).

### 3.7. Effects of CUR Ingestion on GP and GS Activities and Liver Glycogen Levels in Obese Rats

**Figure 5:**
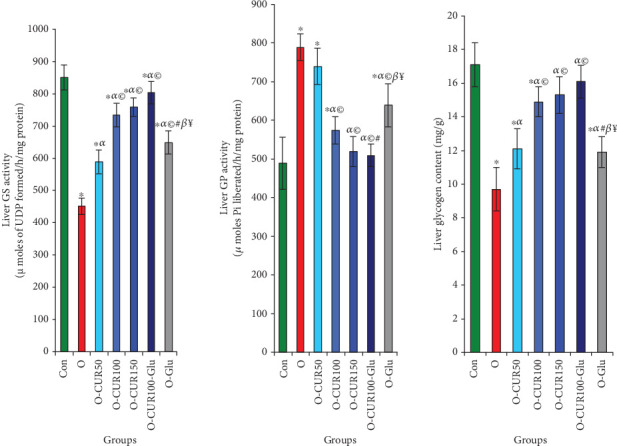
Liver GS and GP activities, as well as liver glycogen rates in obese rats treated with CUR at different doses, are presented. Curcumin administration promotes glycogen biosynthesis by inducing GS and inhibiting GP. Statistical analyses are provided in Figure 2.

Our results indicate that obesity associated with Type 2 diabetes or insulin resistance leads to a reduced capacity to store glucose as glycogen, as demonstrated by a 47% reduction in hepatic GS activity, a 61% increase in GP activity, and a 43% decrease in glycogen levels compared to normal rats. In contrast, CUR supplementation at a dose of 100 mg/kg resulted in a 62% increase in GS activity, a 27% reduction in GP activity, and a 53% increase in hepatic glycogen levels, leading to a reduction in circulating glucose levels (Figure 5).

### 3.8. Effects of Obesity and CUR on Liver Enzymes Involved in Glucose Metabolism

**Figure 6:**
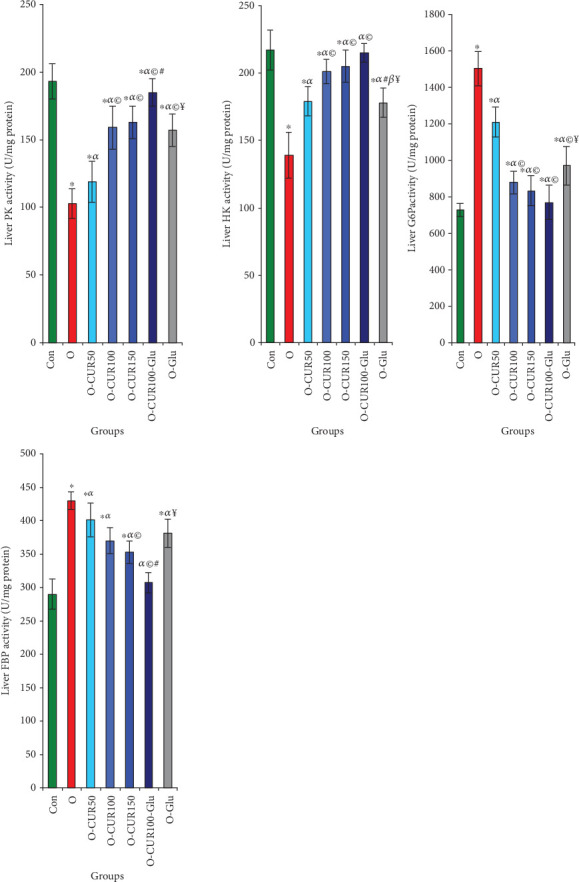
Effect of Type 2 diabetes and CUR on liver glucose anabolism enyme activities (G6P and FBP) and catabolism (HK and PK). Curcumin ingestion by obese rats induces catabolism and suppresses glycemic anabolism. Statistical analyses are provided in Figure 2.

This study reveals that obesity and Type 2 diabetes are associated with increased hepatic activity promoting the release of free glucose, induced by the elevated activity of G6P, which is involved in the final step of gluconeogenesis, where it catalyzes the conversion of glucose-6-phosphate to free glucose, showing a 106% increase. Additionally, the hepatic enzyme FBP, essential in gluconeogenesis, catalyzes the conversion of fructose-1,6-bisphosphate to fructose-6-phosphate, with a 48% increase in activity compared to normal rats. Conversely, CUR ingestion promotes glycolysis and reduces glycogenesis in a dose-dependent manner. Specifically, a dose of 100 mg/kg of CUR stimulates glycolysis, increasing HK and PK activities by 45% and 54%, respectively, while suppressing glycogenesis, as indicated by a reduction in G6P and FBP activities by 41% and 13.9%, respectively, compared to untreated Type 2 diabetic rats (Figure 6).

### 3.9. Obesity, CUR Ingestion, and Oxidative Stress in the Serum of Obese Rats

**Figure 7:**
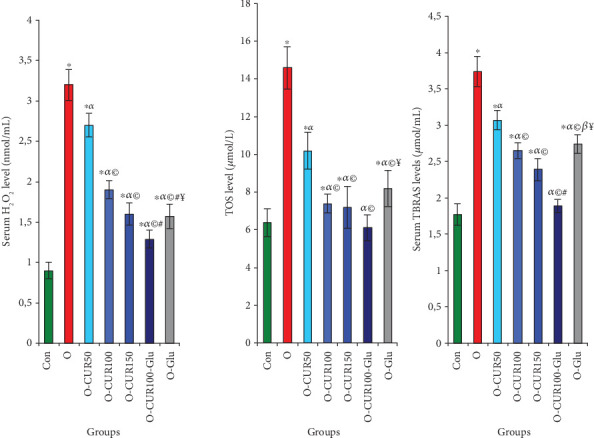
Levels of oxidative stress indices, such as H_2_O_2_ and TBARS levels in the serum of rats with obesity and Type 2 diabetes. Curcumin ingestion was found to protect against oxidative stress, as evidenced by the suppression of the levels of these two indices compared to obese individuals not treated with curcumin. Statistical analyses are provided in Figure 2.

Our results indicate that obesity is associated with a significant increase in H_2_O_2_ levels and oxidative stress markers, including TOS and TBARSs. However, the administration of CUR to obese rats exhibited a strong dose-dependent antioxidant activity, with the most potent effects observed at CUR100 and CUR150 doses. Specifically, the daily supplementation of CUR100-Gluc in obese rats effectively prevented and reduced oxidative stress, protecting tissues from damage. This was evidenced by a 58%, 59%, and 49% suppression of H_2_O_2_, TOS, and TBARS levels, respectively (Figure 7).

### 3.10. Effect of CUR on Obesity-Induced Hyperglycemia, Elevated HbA1c Levels, and Decreased Hemoglobin (Hb) Levels

**Figure 8:**
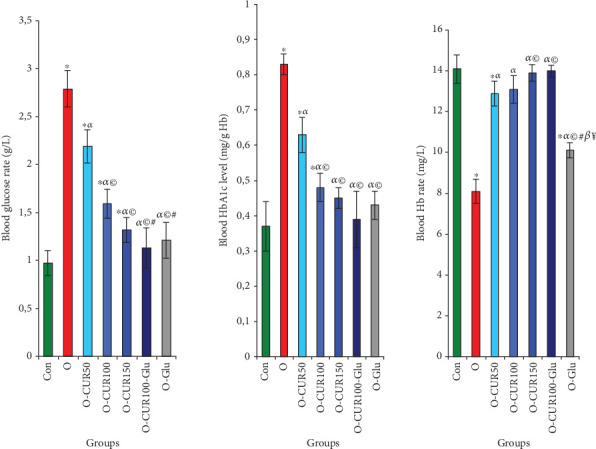
Levels of glucose and glycated hemoglobin in the serum of obese rats with Type 2 diabetes were measured. Our results indicate that obesity significantly increases these two indices. However, administration of curcumin in obese rats significantly reduced blood sugar and HbA1c levels. Statistical analyses are provided in Figure 2.

The results of this study indicate that obesity is associated with elevated blood glucose levels, which promote Hb glycosylation, leading to increased HbA1c levels and reduced Hb levels. However, the administration of isolated CUR normalized these parameters in a dose-dependent manner. Specifically, daily supplementation with CUR100-Gluc significantly reduced blood glucose levels by 59%, consequently lowering HbA1c levels by 53% and increasing Hb content by approximately 73% compared to untreated obese rats (Figure 8).

### 3.11. Obesity, CUR Ingestion, and Systolic Blood Pressure

**Figure 9:**
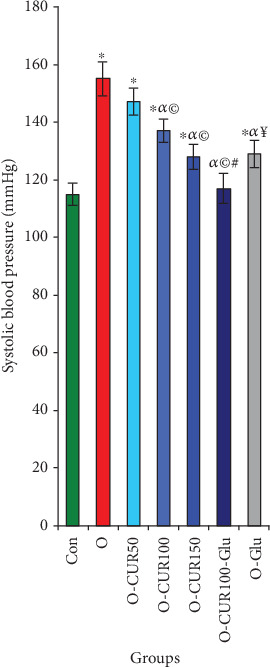
Effect of CUR administration in obese rats with Type 2 diabetes on systolic blood pressure. The results of this study clearly show that the ingestion of CUR at a dose of 100–150 mg/kg is effective in preventing hypertension. Statistical analyses are provided in Figure 2.

The results of this study show that obesity associated with Type 2 diabetes is linked to a significant increase in systolic blood pressure values compared to normal rats (*p* < 0.001). The ingestion of CUR in obese rats led to a dose-dependent decrease in systolic blood pressure. Notably, antihypertensive activity was observed in rats treated with the combinations O-CUR100-Glu and CUR150 alone (Figure 9).

## 4. Discussion

After the extraction and isolation of CUR, FTIR analysis revealed characteristic absorbance bands corresponding to its functional groups. The strong absorbance in the 3600–3317 cm^−1^ range suggests the presence of phenolic hydroxyl (-OH) groups, known for their stretching vibrations in this region. In the aliphatic region, peaks at 2924 and 2854 cm^−1^ correspond to CH₃ and CH₂ stretching vibrations, respectively, indicating the presence of aliphatic chains. The presence of a carbonyl (C=O) group is confirmed by a stretching vibration at 1620 cm^−1^, a key feature of many organic compounds. The aromatic nature of CUR is supported by C=C stretching vibrations observed at 1597, 1504, and 1427 cm^−1^, confirming the presence of an aromatic ring system. Additionally, the strong signal at 1273 cm^−1^, indicative of =CH in-plane deformation, along with the C=O bending vibration around 1195 cm^−1^, further supports its structural identity. The asymmetric and symmetric stretching vibrations of the C-O-C group at 1026 cm^−1^ suggest the presence of ether linkages, which may influence the compound's stability and reactivity. Finally, the out-of-plane bending vibrations of C-H at 964 cm^−1^, as well as the aromatic C-H bending vibrations at 864 and 810 cm^−1^, further confirm the compound's aromatic characteristics. These findings are consistent with the study by Espinoza-Torres et al. [45], which reported similar functional groups and absorbance ranges for CUR. Furthermore, our results align with those of Van Nong et al. [46], who demonstrated that CUR exhibits characteristic absorbance bands at 1625 cm^−1^, attributed to *ν* C=O and *ν* C=C, which are in agreement with our findings. This further validates the efficiency of our extraction protocol. The purity of CUR is also confirmed by absorbance measurement, where we found a maximum absorbance at 427.86 nm, consistent with the literature value of 422 nm [47]. This agreement indicates the presence of conjugated double bonds, a key characteristic of CUR's structure. Our results align with later studies [48–50], which confirm the presence of these functional groups in pure CUR. Specifically, the broad bands for phenolic hydroxyl groups and the identified stretching vibrations for aliphatic C-H, carbonyl, and aromatic C=C bonds are consistent with earlier findings. The UV spectrum's absorption peak also corroborates the literature values for conjugated double bonds [48–50].

The investigation revealed that feeding rats with HFFD for 12 weeks significantly increased the lipase activity in the pancreas and intestine, along with body weight gain, and led to alterations in the serum lipid profile. These findings are consistent with previous studies [51–54] which indicate that a HFFD can induce obesity through various mechanisms. These include increased lipase activity, elevated LDL and TG levels, and decreased HDL-C levels in rats. These changes lead to heightened lipid hydrolysis, absorption, and accumulation, thereby contributing to being overweight and obesity.

A preventive strategy involves inhibiting the absorption of free fatty acids and triglycerides in the small intestine. This approach can help combat hyperlipidemia, hypercholesterolemia, obesity, and coronary artery diseases. Thus, targeting lipase activity in the intestine and pancreas is a promising strategy for managing obesity. This study investigates the effects of CUR administration on obese rats, revealing a significant suppression of both the intestinal and serum lipase activities. This reduction in lipase activity decreases the digestive hydrolysis of lipids, preventing their conversion into absorbable simple fatty acids and triglycerides. As a result, it suppresses the absorption or passage of lipids from the intestines to the tissues (where lipid accumulation occurs). This reduction in lipid accumulation in tissues, or the body, leads to a decrease in body weight by 26.7% after ingestion of CUR at a dose of 100 mg/kg bw, and by about 29% following a dose of 150 mg/kg bw, demonstrating its potential antiobesity effects. These positive effects of CUR on lipase activity are further supported by the notable reductions in LDL-C, TC, and TG levels, as well as an increase in HDL-C levels in the serum of obese rats. These findings align with previous research [55–57], which demonstrated that a diet containing 1% CUR significantly reduced liver triglycerides levels in mice. This suggests that this compound could be an effective therapeutic strategy for managing fatty liver disease associated with hyperlipidemia and obesity, as supported by Wang et al. [58] found that CUR reduces obesity induced by a HFFD. This effect occurs through the modulation of intestinal epithelial cells and the intestinal barrier. Han et al. [59] demonstrated CUR significantly decreased lipopolysaccharide-induced interleukin-1 beta, suggesting that intestinal epithelial cells and the intestinal barrier are key sites of CUR action.

The results indicated that CUR treatment in high-fat diet (HFD)–fed mice reduced body weight gain and improved cold tolerance due to enhanced adaptive thermogenesis [60]. Additionally, CUR improved insulin resistance in fructose-fed rodents and decreased adipogenesis and fat mass in HFD models [61]. A previous study [61] has demonstrated the CUR ability to inhibit adipocyte proliferation and differentiation. Furthermore, CUR promotes the browning of the white adipose tissue in diet-induced obese mice, increasing energy expenditure and reducing fat mass deposition.

This study clearly demonstrated that obesity, characterized by excessive fat accumulation, is associated with insulin dysfunction and the development of Type 2 diabetes. This is evidenced by the increased activity of digestive enzymes such as *α*-amylase, maltase, and sucrase, along with reduced hepatic glycogen levels. Additionally, overweight caused dysregulation of metabolic enzyme activity, characterized by an increase in G6P and FBP, which are involved in glucose synthesis, alongside a decrease in HK and PK activities. This dysregulation led to a reduction in insulin sensitivity, as evidenced by a significant increase in the activity of key enzymes associated with insulin resistance, such as DPP-4 and PTP1B. As a result, blood glucose and HbA1c levels were elevated, while Hb levels were reduced. Conversely, CUR treatment in obese rats, particularly at a dose of 100 mg/kg, led to a dose-dependent reduction in the digestive enzyme activity. The combination of CUR at dose 100 mg/kg with glucor (CUR100-Glu) proved to be the most effective in combating obesity and Type 2 diabetes. This inhibition of digestive enzymes reduces starch, maltose, and sucrose hydrolysis, leading to lower blood sugar levels, consistent with previous studies [62, 63] that have shown inhibition of digestive enzymes to be an effective strategy for the therapy of Type 2 diabetes.

Additionally, CUR intake in obese rats enhanced hepatic GS activity while reducing GP activity in a dose-dependent manner, with the most notable effect observed at the 100 mg/kg dose. This effect may be attributed to CUR insulin-mimetic properties [64–66], which normalize metabolic enzyme activity by suppressing G6P and FBP, thus inhibiting glucose and fructose synthesis. Moreover, CUR promotes carbohydrate catabolism by increasing the activity of HK and PK. These findings align with the study by Seo et al. [67], which reported that CUR's role in glucose homeostasis is mediated through its activation of glycolysis, inhibition of hepatic gluconeogenesis, and reduction of lipid metabolism. Oral CUR supplementation has also proven effective in treating hyperglycemia in genetically diabetic *KK-Ay* mice and streptozotocin-induced diabetic rats [68, 69]. Additionally, research involving HFD-fed hamsters showed that CUR enhanced insulin sensitivity, as evidenced by reduced insulin resistance index values [70].

Interestingly, our study revealed that CUR administration in obese rats activated the insulin signaling pathway by potentially inhibiting the activities of DPP-4 and protein PTP1B. Blocking PTP1B can enhance the insulin signaling pathway, leading to more efficient carbohydrate utilization and glycogen synthesis. Inhibiting DPP-4 may elevate GLP-1 levels, which stimulates insulin secretion and reduces fat and carbohydrate breakdown. Our findings align with those of Ali-Oluwafuyi et al. [71], who showed that CUR induces GLP-1 release from enteroendocrine L-cells, a major source of this antidiabetic hormone. Kato et al. [72] reported that oral CUR enhances GLP-1 secretion, resulting in reduced blood glucose levels through increased insulin secretion. Additionally, Tian et al. [73] demonstrated that administering CUR at a dose of 60 mg/kg to leptin-deficient (ob/ob) mice for 8 weeks increased GLP-1 levels, thereby enhancing energy expenditure and improving both obesity and glucose tolerance.

This study underscores the connection between Type 2 diabetes or insulin resistance and elevated blood glucose levels, which lead to oxidative stress, as evidenced by increased serum H_2_O_2_. The increase in these free radicals causes cellular damage, as indicated by a significant rise in TBARS levels [15]. CUR administration to obese rats mitigates obesity and hyperglycemia, offering a protective effect against oxidative stress. The previous research by Jakubczyk et al. [74] has highlighted CUR diverse properties due to its chemical structure, including antioxidant, anti-inflammatory, and antimutagenic effects, while Memarzia et al. [75] confirmed its potent antioxidant activity. Consistent with these findings, [76] reveals that metabolic syndrome, encompassing obesity and Type 2 diabetes, is linked to various cardiometabolic risk factors, including hypertension. Notably, CUR demonstrated antihypertensive effects in obese rats, aligning with Joshi et al. [77], who found that CUR and its analogues exert antihypertensive effects through multiple signaling pathways.

## 5. Conclusion

Our findings indicate that CUR not only serves as an effective coloring agent in food products but also functions as a therapeutic compound with significant potential for the prevention, amelioration, and treatment of multiple disorders, including obesity and Type 2 diabetes. The mechanisms by which CUR exerts its beneficial effects involve the retardation of carbohydrate and lipid digestion and absorption, as well as the enhancement of glucose catabolism. This is achieved through the suppression of DPP-4 and PTP-1, leading to the induction of insulin signaling. Consequently, CUR demonstrates a significant potential as a functional natural food colorant for the management and mitigation of obesity, Type 2 diabetes, and oxidative stress.

## Figures and Tables

**Table 1:** Effect of CUR on average food and water intake (AFI and AWI), TC, LDL-C, and HDL-C levels in the serum of obese rats.

	**Con**	**O**	**O-CUR **_**50**_	**O-CUR **_**100**_	**O-CUR **_**150**_	**O-CUR **_**100**_ ** -Gluc**	**O-Gluc**
AFI	19.3 ± 1.1	28.1 ± 1.3^∗^	25.3 ± 0.9^∗^^*α*^	22.1 ± 1.1^∗^^*α*©^	21.7 ± 1.2^∗^^*α*©^	20.3 ± 0.6^∗^^*α*©^	22.9 ± 1.3^∗^^*α*©¥^
AWI	20.2 ± 1.5	25.3 ± 1.7^∗^	24.1 ± 0.5^∗^	22.3 ± 1.9^∗^^*α*©^	21.4 ± 0.9^∗^^*α*©^	20.9 ± 1.5^∗^^*α*©^	22.1 ± 1.1^∗^^*α*©^
TC (g/L)	0.93 ± 0.08	1.41 ± 0.16^∗^	1.18 ± 0.05^∗^^*α*^	0.97 ± 0.06^∗^^*α*©^	0.95 ± 0.04^∗^^*α*©^	0.94 ± 0.04^∗^^*α*©^	0.93 ± 0.06^∗^^*α*©^
HDL-C g/L)	0.50 ± 0.05	0.42 ± 0.05^∗^	0.52 ± 0.03^∗^^*α*^	0.58 ± 0.02^∗^^*α*©^	0.59 ± 0.03^∗^^*α*©^	0.6 ± 0.03^∗^^*α*©^	0.54 ± 0.02^∗^^@©#*β*¥^
LDL-C g/L)	0.43 ± 0.04	0.99 ± 0.04^∗^	0.69 ± 0.03^∗^^*α*^	0.48 ± 0.09^∗^^©*α*^	0.45 ± 0.06^∗^^*α*©#^	0.41 ± 0.05^∗^^*α*©#*β*^	0.59 ± 0.05^∗^^@©#*β*¥^

*Note:* The experimental groups are represented as follows: Con refers to healthy control rats, O denotes obese rats, O-CUR50 represents obese rats treated with 50 mg/kg body weight of curcumin, O-CUR100 corresponds to obese rats treated with 100 mg/kg body weight of curcumin, and O-CUR150 signifies obese rats treated with 150 mg/kg body weight of curcumin. Additionally, O-Gluc indicates obese rats treated with glucor. The values are presented as mean ± SD for each group of five animals. Statistical significance is indicated as follows: ⁣^∗^*p* < 0.05 when comparing to controls; ^*α*^*p* < 0.05 when comparing to obese rats; ^©^*p* < 0.05 when comparing to obese rats treated with CUR50; ^#^*p* < 0.05 when comparing to obese rats treated with CUR100; and ^*β*^*p* < 0.05 when comparing to obese rats treated with the combination CUR150; and ^¥^*p* < 0.05 when comparing to obese rats treated with the combination O-CUR_100_-Gluc.

*Abbreviations:* AFI, average food intake; AWI, average water intake; HDL-C, high-density lipoprotein cholesterol; LDL-C, low-density lipoprotein cholesterol; TC total cholesterol.

## Data Availability

The data used to support the findings of this study are available from the corresponding author upon request.

## General Statement

*AI Usage Statement*. No AI software was used in the preparation of this manuscript. All analyses, writing, and editing were conducted manually by the authors.
